# Lectures on Clinical Medicine, Delivered at the Philadelphia Medical Institute

**Published:** 1838-04-25

**Authors:** W. W. Gerhard

**Affiliations:** Physician to the Philadelphia Hospital, &c.


### CLINICAL LECTURES.

### LECTURES ON CLINICAL MEDICINE, *delivered at the Philadelphia Medical Institute, by* W. W. Gerhard, M. D., *Physician to the Philadelphia Hospital*, &c.

#### INTRODUCTORY.

*Thursday, April* 12th. Dr. Gerhard commenced: Gentlemen, a course of lectures on clinical medicine differs from one on theory and practice, in its demonstrative and practical character. It is necessary for a teacher of the practice of medicine to pass through the circle of diseases, describe their forms, their history, and their mode of treatment, in a methodical manner. Such an order is pursued as may be best in accordance with the views of the teacher, but it is always requisite, that he should present to the mind of the pupil, a connected and systematic view of pathology and treatment.

Clinical medicine is the application of the precepts of the art to the recognition and treatment of disease. It requires demonstration in order to point out the phenomena as presented in nature, to accustom the pupil to the varieties they exhibit, and to habituate him to the use of those precautions which are requisite, in order to detect the signs of disease, which would escape a superficial inspection. As the phenomena of disease must be studied, in the order in which they chance to occur to us, a clinical course is less regular in its arrangement, but more precise and exact in its evidence, than one upon the practice of medicine. It requires a sustained attention to reduce to order the assemblage of facts, which are presented in succession, but which are always dependent upon fixed and invariable laws. To trace these laws, and as far as possible to point out the precision to which certain departments of medicine have been carried, and the still more rigorous demonstrations, of which the entire science will be one day capable, is the great object of the present course. In the beginning, many of the relations of disease will escape the attention of the pupil, but, in proportion as his knowledge of nature increases, the beauty and harmony of the laws of pathological action will seem not less striking, than the order which regulates the healthy actions of the economy. A single disease will then, in a great measure, include a knowledge of all others, for it is the comparison of the symptoms of disease, which enables us to distinguish one from the other; it is the knowledge of the relations which they mutually bear to each other, which enables us to adopt a successful curative or prophylactic practice.

There is but one method of becoming familiar with these truths; it is the frequent and repeated examination of them, from different points of view; if the results thus obtained agree one with another, the deduction assumes the force of a mathematical demonstration, if they differ, we may be sure that the error lies in our powers of observation, or in our imperfect reasoning, which prevents us from appreciating the regular and fixed laws of nature. It is now well known, that acute articular rheumatism is a frequent cause of chronic diseases of the heart. This fact is proved in two ways; first, by direct observation of patients affected with a rheumatism in whom an acute disease of the heart appears, which assumes a chronic form, after the affection of the joints has entirely disappeared, and secondly, by carefully interrogating patients who labour under chronic disease of the heart, which has evidently dated from an attack of rheumatism, which had occurred long before. This is one of the simple illustrations of our meaning; in the progress of the course, other and more complicated instances will be brought forward, which exemplify the laws of relation, in a still more beautiful mannner.

A knowledge of the relations of disease necessarily requires a familiarity with diagnosis, or the art of distinguishing one disease and one variety of disease from another. Diagnosis, on the other hand, requires a knowledge of semeiology, or the art of recognising the signs of disease, by the alterations which they produce in the functions of the body, and of connecting these altered functions or symptoms with the changes in the condition of the organs to which they correspond. As the important organs of the body are concealed from our direct observations, we become aware of the changes which occur in them during life, by an examination of the traces of diseased action which are left after death. The investigation of the disorders found after death, their classification and connexion with changes occurring during life, constitute the science of pathological anatomy.

These various branches of medical science are so intimately connected with clinical medicine, that they form, as it were, a necessary part of it, and are indispensably requisite for the pupil. For convenience of study, they may be learned separately. In this course, they will be taught in such a manner as not to interfere with the regular demonstrations of disease; a part of the course being devoted strictly to clinical medicine, and a part to the acquisition of those means of investigation which the physician requires, in order to become familiar with disease. By directing your attention to the study of disease, at the same time that you are learning the arts necessary for its exploration, you will find, that practice will render theory precise, and strip it of what is obscure and doubtful.

It is not necessary to enter into complete details of the symptoms and treatment of each disease; I shall, however, in all cases, point out the treatment which is certainly known to be effectual in arresting the disorder, and distinguish it from therapeutics, which rest upon probable evidence. Where the best treatment in a case cannot be positively known, I shall indicate the probable success of particular methods. In those diseases which have a natural duration, treatment has much power in diminishing the severity of the disorder, and in lessening its mortality, but it does not suddenly arrest the symptoms. This class of disorders is apt to lead the practitioner into error, at the beginning of his career, and furnishes the most common pretext for the success of charlatans. It is, therefore, the duty of a clinical teacher to distinguish them carefully from other affections, and to analyze the exact power of remedies.

At my next lecture, I shall enter upon the regular course, and commence with the subject of acute articular rheumatism. In proportion as we advance in our study of disease, the mode of investigation, which I have just pointed out, will become more intelligible, and of more easy application.

NOTE.—This course of lectures will be continued during the summer, in as regular an order as the nature of the subject will permit. Reports of them will be given in the Examiner.

---

### ACUTE ARTICULAR RHEUMATISM.

*Tuesday, April* 17th. Dr. GERHARD remarked: I shall to-day, gentlemen, take up the subject of acute articular rheumatism, as it is especially prevalent at this season of the year, when the number of other acute diseases is very limited. In summer we have acute abdominal inflammations, and in winter affections of the pulmonary organs, while, during the spring and early summer months, serous inflammations, both of the internal and external membranes, are most common. Acute rheumatism bears some striking relations with the inflammations of internal serous membranes, from the similarity of the mode of treatment which often becomes necessary in both affections, and from the frequent complication of the latter with the former disease. In almost every severe case of the disease under notice, there co-exists inflammation of the covering of the heart, or of the serous membrane lining its cavities. Since this connection between the pathology of articular rheumatism and that of diseases of the heart and its membranes, has been clearly traced, the disease has attracted much interest. I say clearly traced, for the general fact had been long since pointed out, although the subject was not precisely understood. That is, it was in the same situation as many other parts of pathology; affording an indistinct view of the truth, but without that well defined character which is now required, according to the rules of rigid logic applied to the study of pathology. The line of connection has only been drawn in a definite manner, for a few years past, between acute articular rheumatism, and endocarditis, or inflammation of the lining membrane of the cavities of the heart, and pericarditis, or inflammation of the membrane surrounding it. Dr. Bouillaud, of Paris, has paid particular attention to this subject; he tells us that at least one-half the acute articular affections are complicated with pericarditis. In this estimate he is probably wrong, unless with pericarditis we are permitted to include endocarditis. A large number of mild cases doubtless run their course, without any complication of the kind, but it is usually otherwise when the disease appears under a severe type. Mild cases are slow in their action upon the heart, but, in the severer forms, the advance is rapid, and disease of the heart succeeds almost immediately after the first appearance of the articular symptoms. In chronic cases, the progress of the cardiac affections is slow, and an individual not well acquainted with the disease might be deceived as to the fact of its existence.

We have thus traced two forms of the disease, and I propose bringing under your notice two cases now under treatment in the Philadelphia hospital, to exemplify them.

The first is that of John Robb, who was admitted into the ward No. 2, on the 11th of April. Previous to his admission, he had been ill but a short time, being an inmate of the Alms-House, he was able to resort to medical assistance, quite as soon as is usual in private practice. He had been working on the farm of the establishment for eight months previous to his attack, and had enjoyed good health. On the morning of the sixth, he complained of slight pains in his shoulder, but continued at work; at eleven, P. M., he was taken with severe pain in the hip, which lasted four or five hours, and then, diminishing in the hip, went to the knee. On the seventh it ceased in the right knee and went to the left.

Now, from the character of the affection, thus shown, we can, without going farther, make our diagnosis. I allude to its metastatic character, as exhibited in its leaving one joint and settling in another. This is distinctive of rheumatism.

There was no pain in the ankles, but there was slight pain near the toe. You here mark the course of the disease onwards; it has reached the toe, and, showed a disposition to attack the foot. There had been pain in the right wrist, from an hour before the man's entrance into the hospital. On the ninth and tenth, he had pain in the breast, which he referred to a spot below the præcordia, in the region of the diaphragm, and which lasted twenty-four hours and was increased by coughing. Such pain is usually owing to disease of the heart, which may be merely muscular, but is more frequently caused by inflammation of the serous membranes, lining or covering the heart.

This man had been exposed to no causes of disease, other than those which he was in the habit of encountering. He had, it is true, been wet while working on the farm, but this was not uncommon with him; he had been long accustomed to working in the rain. This shows how cautious we should be in admitting causes of disease; some physicians might be disposed to attribute the attack of rheumatism to the last wetting, which could manifestly exercise no greater influence upon the man than a series of previous exposures to the same cause, of no recent standing. I look upon the particular season of the year, as the immediate excitant of the disease, and it is for this reason, that I have thought it a fitting subject to bring before you, at the opening of my course. If you take the trouble to inquire, you will find, that at this time the prevalence of rheumatisms and rheumatic pains is remarkable.

The case before you being of an acute character, its previous history is not nearly so important as the present state of the individual. It is otherwise in chronic affections, in which the whole anterior history is all-important.

The condition of the patient, at the time of his admission on the eleventh, was as follows. The face was slightly flushed, and presented an expression of pain. This pain, in acute rheumatism, is remarkable; it usually prevents all exercise and confines the patient to bed.

There was slight soreness in the shoulder, but without swelling or heat; no pain or swelling in the left arm, slight soreness in the right elbow and severe pain, swelling and heat in the wrist. The same pain, swelling, and heat extended to all the joints of the hand and fingers, excepting the thumb. There was some pain in both knees, especially in the left; none in the ankles; a little in the right hip, no tenderness of the spine, no cephalalgia; tenderness on pressure along the region of the ribs; this was probably the remains of the diaphragmatic pleurisy. The impulse of the heart was feeble, the second sound nearly lost, the first much roughened, a dulness on percussion nearly natural. Treatment, one grain of opium every four hours. The digestive organs were healthy.

Now, let us analyze this case. The first fact worth recollecting, is the absence of tenderness of the spine. This establishes the diagnosis between rheumatism and neuralgia. Hence, the mode of treatment which proves so excellent in the latter affection, may here fail. The state of the heart indicated merely slight valvular disease, and some muscular impediment; there was no effusion, the dulness on percussion being natural, and no creaking sound being heard.

The treatment in this case was after a plan of practice in New England, from which quarter it has been lately strongly recommended; the internal administration of opiates, pushed till felt by the patient.

During the twelfth, six grain pills of opium were exhibited, but there was no diminution of pain. Neither sleep nor cephalalgia had been induced by the opium. This is an important therapeutic point, demonstrating the antagonising action which pain exerts in regard to the effects of opium. The first sound of the heart was still rough, but the impulse rather less; no increase of flatness. The state of the heart was, therefore, slightly improved. Pulse eighty-four, of moderate size and regular; a grain of opium was ordered every two hours, and a laxative enema administered.

On the evening of this day, there was some cephalalgia, although no deviation of the pupils from the natural state. The dose of opium was diminished to a grain every three hours. Sleep was interrupted by twinges of pain; sweating at night. Eruption of sudamina; pulse seventy-two; pain in right arm increased and extending to the shoulder. Less pain and swelling in the knees, but increase of both in the feet. The action of the heart was more regular and feeble, and the sound less rough. The disease, you perceive, was not in any manner arrested, although you note a decided improvement in the condition of the heart. There was costiveness from the opium, but this, you will soon see, disappeared. Same prescription of opium continued during the thirteenth; hop poultices to most of the painful joints; laxative enema.

On the fourteenth, the pain having diminished throughout the right arm, began in the left hand and wrist. Here is another point of interest; the translation of the pain from the right to the left limb by metastasis. This is a common thing in articular rheumatism, and, as in this case, the pain does not usually quite cease in one joint, before it begins in the other.

There was slight pain between the shoulders, and diminution of the pain in the knees and feet; pulse seventy-six, fuller and regular; this is somewhat an exception to its usual condition in the disease, it being frequent, small, and quick. Skin warm and dry; sleep very irregular; tongue moist, with a yellowish coat; appetite bad; thirst; three or four stools since the enema; the opium had, therefore, induced no costiveness. No cephalalgia or dizziness; slight flush; eyes natural. Opium continued, hop poultice and laudanum to the left wrist.

On the fifteenth, the left hand was worse, and there was pain in the sole of the right foot. The other pains were better, moisture rather than sweating. Opium continued.

The sixteenth, less expression of pain, and less flush; soreness in both shoulders, with slight swelling, but not much constant pain. Slight soreness of the left elbow; much swelling, pain, and heat of the left hand; right hand nearly free from swelling, still slightly painful, but motion returned; pains much diminished in the legs; pain at the ensiform cartilage; palpitations frequent after slight exertions; pulse seventy-two, and soft; decided roughness, almost rasping, in the first sound of the heart, which was not very loud, and heard most distinctly to the left of the nipple, second sound nearly lost. Under the sternum, both sounds of the right side distinctly heard and clear, the first only a little roughened. The præcordial dulness commenced only at the left margin of the sternum, and extended to the nipple. The morbid alteration was therefore confined to the left side of the heart, implicating the valves; there was besides effusion into the pericardium. The opium pills were continued during yesterday and last night every three hours. Hop poultices.

Last night, the pupils were somewhat contracted, and little sensible to the light. To-day, the face was flushed, and presented an expression of stupor. Disposition to sleep; pupils rather large; no cephalalgia; sleep interrupted by pain, shooting from the swollen joints. Pulse eighty-eight—softer. Swelling less marked in the left hand. Slight swelling and pain in both knees. No pain in the breast. Impulse of the heart almost lost; both sounds very feeble, without roughness. The disease of the valves is therefore diminished. Percussion slightly dull, at the upper portion of the left side; flat, down from the third rib to the same extent as yesterday. Prominence obviously increased. These latter signs are explained by the increased effusion into the pericardium. Still slight diarrhœa; three or four stools in the twenty-four hours. Skin moist, without swelling. A grain of opium every four hours.

This case, gentlemen, of acute disease of the heart, occurring in articular rheumatism, may serve as a type of the affection, which I shall now make the subject of some general remarks, and have occasion to refer to hereafter. There are several peculiarities to be alluded to. In the first place, the changeable character of the affection, shifting, as you have seen it, from joint to joint, denotes the nature of the disease. This is well understood, and universally admitted. But I would have you remark, that there was no *metastasis* to the heart. The disease of the heart appeared, during the most acute stage of the rheumatic fever, which afterwards continued with unabated severity. By physical examination, we ascertained that the pain in the præcordial region proceeded, first, from disease of the valves, indicated by the roughness of the sound; secondly, from effusion, shown by the unnatural dullness on percussion, imperfect action of the heart, &c.

Another symptom to be noticed, is the sweating, which was very slight from the first or second day, although it is generally very severe in acute rheumatism. It is this sweating in rheumatism, which has suggested the employment of Dover's powder, and other sudorifics, in its treatment. In this case, opium was alone resorted to, to afford a better test of the powers of the remedy.

The diarrhœa is another feature worth remarking, co-existing, as it did, with the large doses of opium. It was a purely accidental complication, but its occurrence demonstrates that opium, in very large doses, does not produce the same effects as in ordinary doses, thus illustrating a therapeutic law, that remedies, in over doses, do not act upon the system in the same manner, as when administered in the usual quantities. Were it not for this law, patients would die from the action of certain remedies now frequently prescribed. How could tartar emetic be given in the high doses required by the contra-stimulant practice, in pneumonia, or calomel, as it is prescribed in the diseases of certain sections of our country?

The pathology of the disease under consideration is still very obscure, although its symptoms are well understood. How much of the disease is like neuralgia, or connected with an affection of the nervous system, and how much belongs to local inflammation, are points that are still unsettled. It is, in this repect, analogous to hooping-cough, and some other diseases. We are just as much in the dark, as to effectual curative means for arresting the progress of the affection, though we have any number of palliatives. For the present, I refrain from expressing an opinion as to the pathology of rheumatism, but shall consider it partly as nervous and partly as inflammatory in its character. Certain inflammation of internal organs which occur in rheumatism, such as pericarditis, lose this doubtful character, and become decided phlegmasiæ; they are accordingly treated without reference to the disease of the joints. When the complication of pericarditis proves fatal, and the opportunity, otherwise rare, of examining persons who die with rheumatism, is obtained, there is almost a total absence of lesion in the joints; but the pericarditis offers the same characteristic appearances, as if it had been induced by exposure to cold, or injury, or some other ordinary cause. The affection of the joints depends so much on a nervous cause, that it presents very slight traces of inflammation. It never terminates in suppuration, or the other usual terminations of inflammation. Dr. Chomel states, that pus is not found in rheumatic joints; the very rare cases in which it is met with, he considers to be mere accidental complications. This opinion, if somewhat modified, is probably the correct one; that is, rheumatic differs from ordinary inflammation in the absence of pus, and its want of fixedness of position. Not so with the accompanying internal inflammations; they result in the secretion of pus, and effusion of lymph, and are fixed in their locations.

If the pathology of the disease is obscure, equally so are the therapeutics, it being more than doubtful that we possess any exclusive available method of treatment. This subject is very clear, no doubt, to some authors; but, unfortunately, practitioners generally are in the dark. Thus Bouillaud, who regards the affection as merely inflammatory, depletes to the utmost possible extent; and for this exaggerated depletory practice claims great success. His success may have been great, but others who have followed the practice, perhaps without the same enthusiastic confidence, have not been so fortunate. I have given the practice a very fair trial, with every disposition to see it succeed, and, although I have afforded relief by one or two moderate bleedings, if persisted in, the result was unfavorable; if pericarditis was present, it was only partially relieved, while the rheumatic affection of the muscular substance of the heart always increased. We thus merely return to the old practice of one or two bleedings at the commencement of the affection; a practice, the utility of which is sanctioned by long experience.

Another practice, originating, I believe, in New England, and recommended by Dr. Webb, of Providence, is that which has been followed in the present case—consisting in the administration of very large doses of opium. I have tried it in two cases, in both of which it failed. It succeeded in stupifying the patient, and rendered him less sensible of pain, but produced no decided impression on the disease. It did not prevent the change of place, nor did it remove the pain or swelling. These symptoms persisted, and retained their usual mutability of character. Last summer, I pushed the remedy to such an extent as to induce decided narcotism, yet I failed to cut short the disease. The remedy may occasionally obtain the success which is claimed for it, but it is clearly no specific.

Sudorifics are the treatment adopted by some, from a notion that artificial sweating is but an imitation of the curative process of nature. This is certainly not the case; for the sweating is most profuse, while the violence of the disease is persisting. If, however, this discharge be suppressed, from cold or any other cause, it will be proper to resort to sudorifics, to revive this natural secretion, and restore to the patient what he has been deprived of.

Other remedies have been recommended, as narcotics and purgatives, particularly the colchicum, and, what is analogous to it, the veratria. The colchicum is used in this country and in England, but is not much employed in France. It is very useful as a palliative, though far from being absolutely curative. I have seen it stop the severer symptoms of the disease, for as much as five or six successive days. I use it at the hospital, in an uncombined form, preferring, as I do, the administration of simple remedies, particularly in hospital practice, to ensure their accuracy of administration, and to enable us to judge of their effects. By giving the wine of the roots or seeds, alone, we may avoid the severe purgation resulting from Scudamore's mixture. Purging may be of service, if the patient can readily bear the motion necessary for the evacuation of the bowels. But the disadvantage, attending frequent rising, is apt to more than destroy the good, arising from the revulsive effects of the purging. In medicine, as well as in surgery, inflamed parts must be kept at rest.

These views, as to the effects of remedial agents in rheumatism, differ but little from those of Dr. Chomel, who, perhaps, has more than a due share of scepticism, in relation to therapeutics. It is true, however, that, when diseases, after running a certain course, get well of themselves, they are apt to deceive us as to the value of the remedies employed in treating them. This, I think, is the case with Drs. Bouillaud and Webb. For the opinions of the former of these physicians on this subject, I refer you to the Select Medical Library; and for Dr. Webb's, to the Boston Medical and Surgical Journal, for last year.

I am not disposed to enter into a history of all the different sorts of medications, which have been recommended in rheumatism. Of external applications, cups to the spine, as a counter-irritant, is a most valuable palliative; and, if the neuralgic element of the disease predominates, cupping along the spine will sometime produce a real arrest of rheumatism. But, when the joints are the principal seat of the disease, in most cases much is not to be expected from cups or leeches to the spine; they do better near the joints. Other applications to the parts are directed for the benefit of moisture and warmth. For this purpose, anodyne poultices are useful; none are better than one of hops, steeped in hot water, or vinegar and water, sprinkled with a little laudanum. These are very convenient applications, but cannot be accommodated to all the joints. Opiate frictions may be used, as with a mixture of warm oil and laudanum. I refrain from lead-water, or spirituous, or other stimulating embrocations, as the danger of the internal affections, endocarditis, or pericarditis, is somewhat increased by driving the affection from the joint. This practice must be reserved for the sub-acute variety of the disease.

Other local applications of a soothing character may be resorted to, such as the experience of every practitioner will suggest. In the North, there are other remedies, the virtues of which are much extolled, such as the green hellebore, actæa racemosa, &c. These plants have been tried here, but without the success which is claimed for them. This want of success may depend on our obtaining them only in the dry state, in which their virtues are impaired. But I cannot believe that this is the sole cause of failure; for the most decided action of the remedies will sometimes be produced, without curing the disease.

Though not immediately dangerous, few affections are ultimately more mischievous than acute rheumatism. Diseases of the heart are so apt to originate with it, and to continue after its cessation, that we must hail any plan of treatment, likely to exercise a curative influence over it. I have therefore tested the opiate practice, as the last which has been recommended, watching very carefully its effects. I certainly pushed it as far as was prudent; I was not warranted in giving more than one grain every two hours, particularly, as I could not see the patient after each dose, a precaution which is always advisable when giving high doses of opium.

In other cases of the disease, I am willing to try other modes of treatment, which are highly recommended, although I fear that they are all merely palliative, and as such only may do good; at last, we may find some one more efficacious than the others. I am doubtful as to immediate success, though strong in hope. I cannot help agreeing with Chomel, sceptical as he is generally, in believing inflammatory rheumatism an affection not to be cut short by remedies, after having seen so much protracted suffering from it; even in the case of physicians, who were treated under the most favorable circumstances, it has been prolonged to four or five weeks and upwards.

What is the natural duration of acute rheumatism? It is not precisely fixed, but is scarcely ever less than two weeks, and may last for five or six months; at least, the immediate effects may continue so long. Like most diseases, that run a determined course, it averages two or three weeks.

Of the second patient, whom I mentioned, my time will allow me to say little or nothing. He offers signs of disease of the heart, different from the last, chronic dilatation and slight hypertrophy, without disease of the valves, the sounds not being at all roughened. There is effusion into the pericardium, indicated by the increased dullness or percussion. Further details I reserve for another occasion.

